# Role of erythropoietin in the angiogenic activity of bone marrow endothelial cells of MGUS and multiple myeloma patients

**DOI:** 10.18632/oncotarget.7587

**Published:** 2016-02-22

**Authors:** Aurelia Lamanuzzi, Ilaria Saltarella, Arianna Ferrucci, Roberto Ria, Simona Ruggieri, Vito Racanelli, Luigia Rao, Tiziana Annese, Beatrice Nico, Angelo Vacca, Domenico Ribatti

**Affiliations:** ^1^ Department of Internal Medicine and Clinical Oncology, University of Bari Medical School, Bari, Italy; ^2^ Department of Basic Medical Sciences, Neurosciences and Sensory Organs, University of Bari Medical School, Bari, Italy; ^3^ National Cancer Institute “Giovanni Paolo II”, Bari, Italy

**Keywords:** angiogenesis, erythropoietin, endothelial cells, monoclonal gammopathy of undetermined significance, multiple myeloma

## Abstract

Increasing evidences suggest several biological roles for erythropoietin and its receptor (Epo and EpoR), unrelated to erythropoiesis, including angiogenesis. Here, we detected the expression of EpoR in bone marrow-derived endothelial cells from monoclonal gammopathy of undetermined significance (MGUS) and multiple myeloma (MM) patients (MGECs and MMECs, respectively) and assessed whether Epo plays a role in MGECs- and MMECs-mediated angiogenesis. We show that EpoR is expressed by both MGECs and MMECs even though at a higher level in the first ones. Both EC types respond to rHuEpo in terms of cell proliferation, whereas other responses, including activation of JAK2/STAT5 and PI3K/Akt pathways, cell migration and capillarogenesis are enhanced by Epo in MGECs, but not in MMECs. In addition, the conditioned media of both Epo-treated cells induce a strong angiogenic response *in vivo* in the chorioallantoic membrane assay, comparable to that of vascular endothelial growth factor (VEGF). Overall, these data highlight the effect of Epo on MGECs- and MMECs-mediated angiogenesis: MGECs are more responsive to Epo treatment than MMECs, probably because over-angiogenic phenotype of MMECs is already activated by their autocrine/paracrine loops occurring in the “angiogenic switch” from MGUS.

## INTRODUCTION

Endothelial cells (ECs) isolated from the bone marrow of multiple myeloma patients (MMECs) express EC markers, including Tie2, vascular endothelial growth factor receptor-2 (VEGFR-2), fibroblast growth factor receptor-2 (FGFR-2), CD105-endoglin and vascular endothelial (VE)-cadherin. Furthemore, MMEC *in vitro* and *in vivo* angiogenic activity is enhanced by an increase in matrix metalloproteinase-2 and -9 (MMP-2 and MMP-9) secretion and by the up-regulation of angiogenic-related genes [1].

Erythropoietin (Epo) is a pleiotropic cytokine that exerts different biological effects, and angiogenesis is one of its extra-hematopoietic functions [2]. Epo and Epo receptor (EpoR) are expressed in the vasculature during embryogenesis, and their deletion in null embryos leads to angiogenic defects [3]. In post-natal life Epo stimulates both proliferation and migration of human and bovine ECs *in vitro* as well as in the rat aortic ring model [4–8]. Moreover, Epo induces endothelin-1 (ET-1) expression in EC cultures [6, 9], and recombinant human Epo (rHuEpo) induces an increased proliferation, MMP-2 expression and differentiation into vascular tubes of human ECs *in vitro* [7]. EpoR mRNA is expressed in different ECs [4, 8].

This study was designed to determine the effects of Epo on ECs from monoclonal gammopathy of undetermined significance [MGUS, (MGECs)] and MMECs in *in vitro* and *in vivo* experimental assays.

## RESULTS

### RHuEpo proliferation of BMECs expressing EpoR

**Figure 1:**
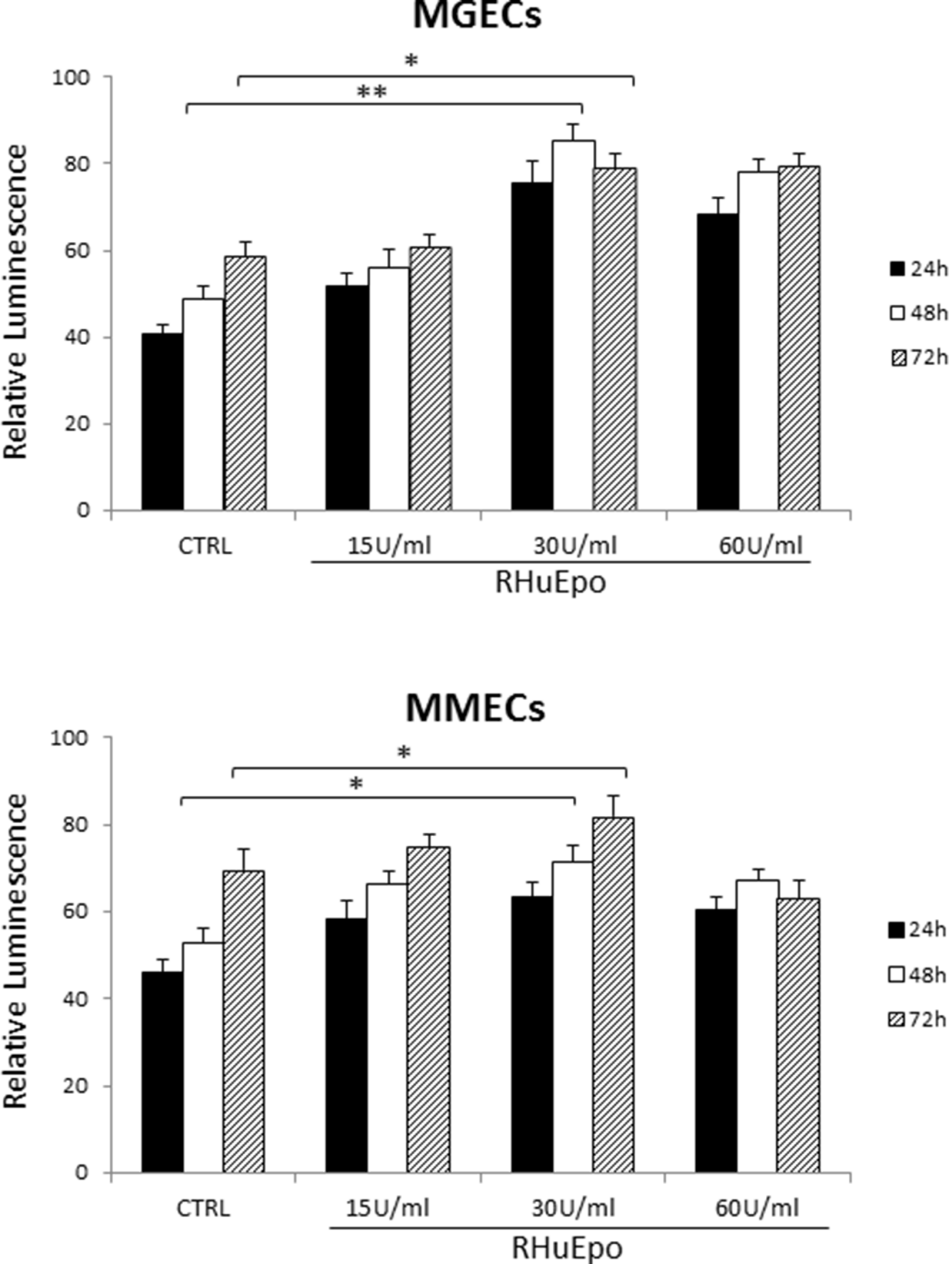
RHuEpo promotes bone-marrow endothelial cells proliferation Cell proliferation assay using CellTiter-Glo^®^ Luminescent Cell Viability Assay of 5 MGECs versus 9 MMECs (as mean ± SD).

**Figure 2:**
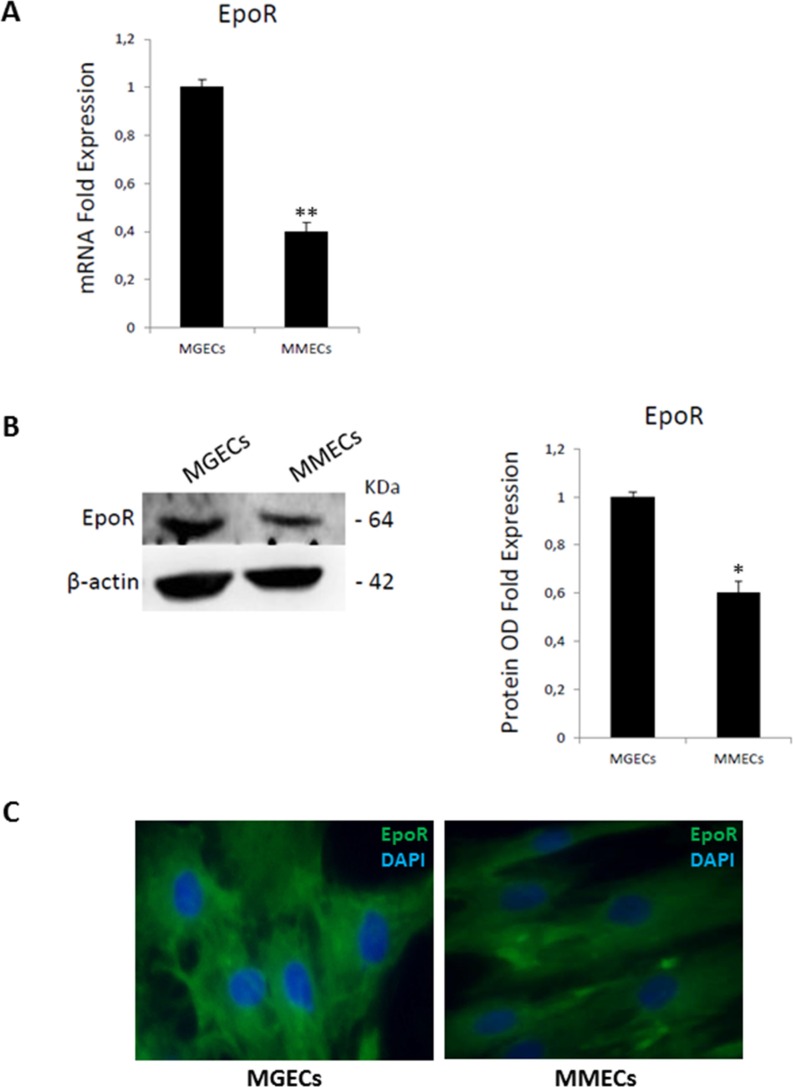
Bone marrow endothelial cells express Epo receptor (**A**) mRNA levels are analyzed by Real Time RT PCR and normalized to GAPDH; fold increase of mRNA in MGECs versus MMECs as mean ± SD of 6 MGUS and 8 MM patients. (**B**) Western blot of representative MGECs and MMECs (left); fold increase of optical density (OD) in MGECs versus MMECs as mean ± SD of 8 MGUS and 12 MM patients (right). Significances *P < 0.03 and **P < 0.003 by Wilcoxon signed-rank test. (**C**) Immunofluorescence for Epo-R (green signal) and nuclei (blue signal) in ECs from representative MGUS and MM patients. Merge show more cell surface expression of Epo-R in MGECs versus MMECs. Left panel: merged picture of Epo-R and nuclei in MGECs; right panel: merged picture of Epo-R and nuclei in MMECs. Pictures acquired by an Axioplan-2 microscope. Original magnification 40X.

To assess the mitogenic ability of rHuEpo, MGECs and MMECs were treated with 15 U/ml, 30 U/ml and 60 U/ml rHuEpo for 24, 48 and 72 h. The addition of rHuEpo significantly increased cell proliferation in a time and dose manner, and the highest proliferative rate was obtained for both MGECs and MMECs at a concentration of 30 U/ml for 48 h and 72 h, (Figure 1). In order to evaluate EpoR expression in MGECs and in MMECs, we performed a Real-Time RT PCR and a Western blotting analysis. Real-Time RT PCR demonstrated that MGECs had a higher expression of EpoR than MMECs (Figure 2A). Western blotting analysis as well as immunofluorescence staining confirmed these data (Figure 2B and 2C).

### RhuEpo regulates secretion of pro-angiogenic factors by BMECs

**Figure 3:**
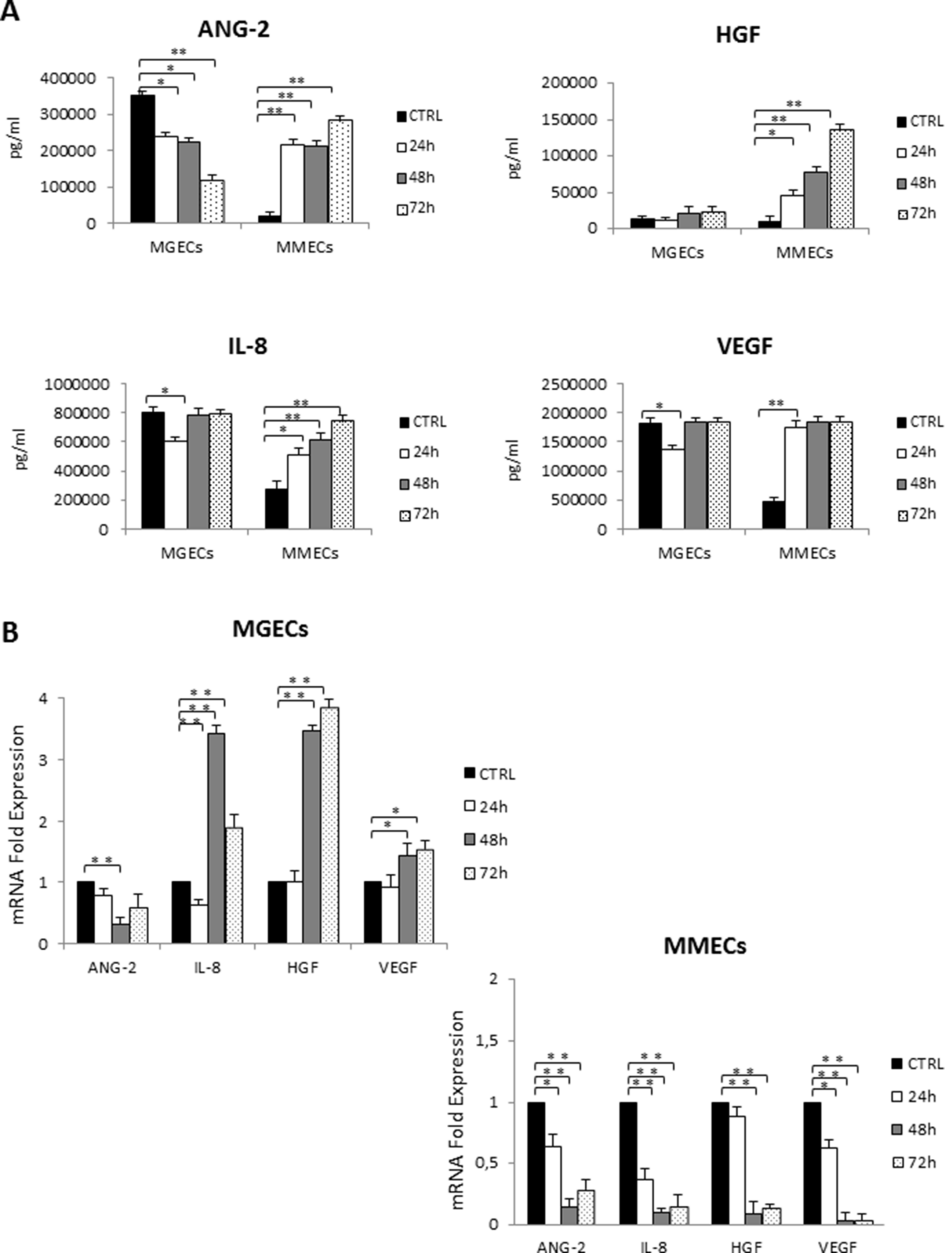
RHuEpo regulates secretion of pro angiogenic factors by bone marrow endothelial cells (**A**) Secreted ANG-2, HGF, IL-8 and VEGF levels quantified by multiplex ELISA using Q-Plex^™^ Array Human Angiogenesis Antigen in conditioned media (CM) of 7 MGECs and 5 MMECs treated with rHuEPO for 24, 48 and 72 h; histograms are expressed in pg/ml. (**B**) mRNA levels of the same cytokines are analyzed by Real Time RT PCR and normalized to GAPDH; fold increase of mRNA in MGECs versus MMECs as mean ± SD of 7 MGUS and 5 MM patients. Significances *P < 0.03 and **P < 0.003 by Wilcoxon signed-rank test.

To evaluate if rHuEpo could modify the angiogenic cytokine secretion, we carried out a multiplex ELISA testing CM from MGECs and MMECs treated with 30U/ml of rHuEpo for 24, 48 and 72 h. ELISA revealed that ANG-2 secretion gradually decreased in MGECs, but it increased in MMECs. HGF was not secreted by MGECs, while its level significantly increased in MMECs in a time-dependent manner. Similarly, IL-8 and VEGF levels were not modified by rHuEpo treatment in MGECs but in MMECs IL-8 release increased in a time-dependent manner and VEGF secretion raised at 24 h as well as at 48 and 72 h (Figure 3A).

Real-Time RT PCR experiments were performed. In MGECs an inhibition of ANG-2 gene expression occurred; IL-8 mRNA levels decreased at 24 h and increased at 48 and 72 h, as well as HGF and VEGF (Figure 3B). On the contrary, in MMECs Real-Time RT-PCR experiments showed a decrease in gene expression of all the pro-angiogenic genes (Figure 3B).

### Stimulation of EpoR elicits activation of JAK2/STAT5 signaling pathway in BMECs

**Figure 4:**
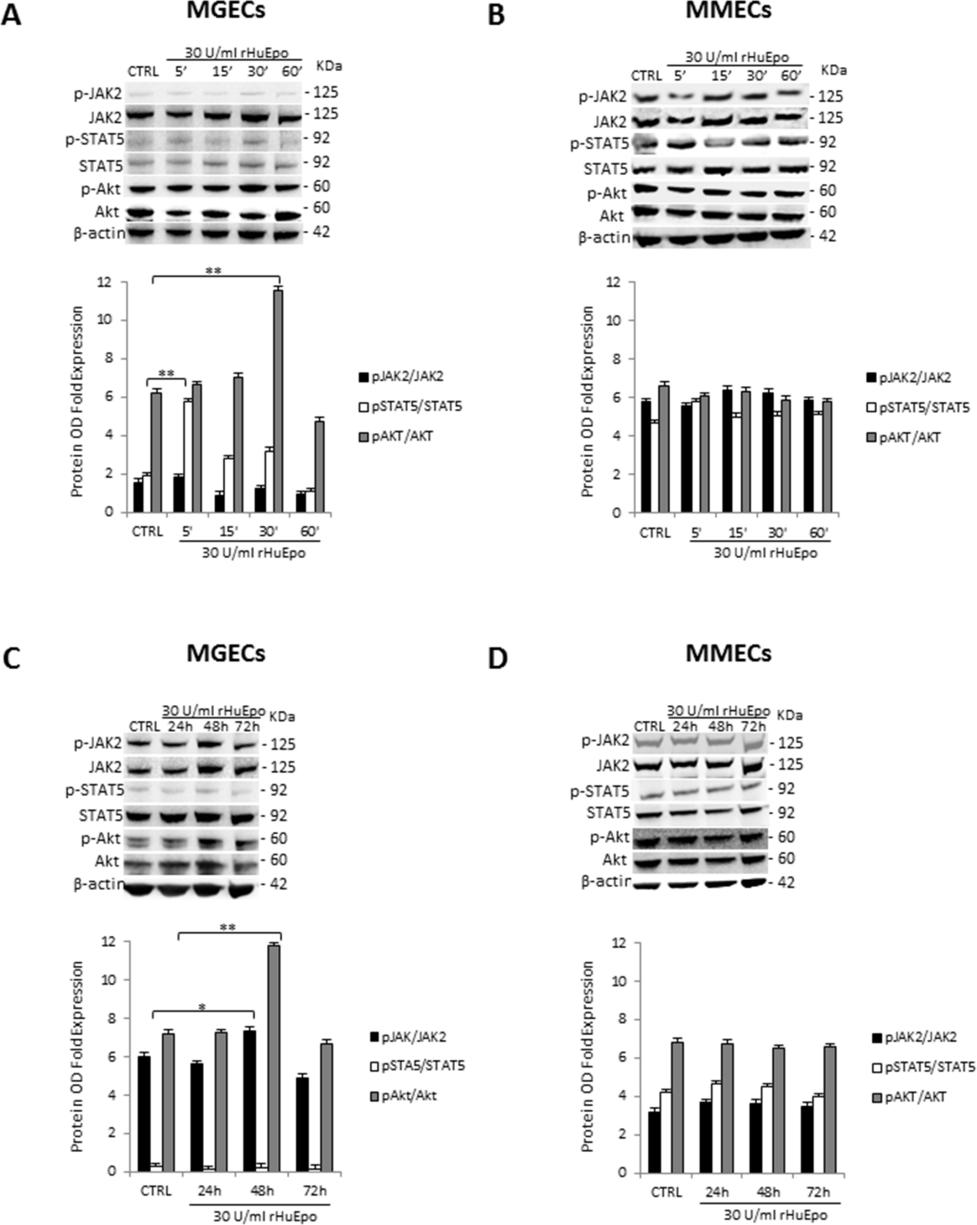
Stimulation of EpoR elicits activation of JAK2/STAT5 signaling pathway in bone marrow endothelial cells Western blot of representative MGECs and MMECs rHuEPO-treated for 5′, 15′, 30′ and 60′ (**A** and **B**) and for 24, 48 and 72 h (**C** and **D**). Fold increase of optical density (OD) of phosphorylated JAK2, STAT5 and Akt expressed as mean ± SD of 4 MGUS and 6 MM patients. Significances *P < 0.03 and **P < 0.003 by Wilcoxon signed-rank test.

To evaluate time correlation of pathways activated by rHuEpo, we have studied the phosphorylation of Epo downstream effectors JAK2, STAT5 and Akt in both MGECs and MMECs (Figure 4). In MGECs rHuEpo triggered an increase of Akt phosphorylation on Ser473 after 30 min of treatment and of STAT5 phosphorylation after 5 min, returning to basal levels after activation. while No change in JAK2 phosphorylation was observed (Figure 4A). Instead, a 48 h treatment led to an increase of JAK2 phosphorylation and Akt activation, but not of STAT5 (Figure 4C). On the other hand, in MMECs RHuEpo did not induce significative modulation of JAK2, STAT5 and Akt phosphorylation at treatments neither short nor long-term (Figure 4B and 4D).

### RHuEpo treatment enhances BMECs motility *in vitro*

**Figure 5:**
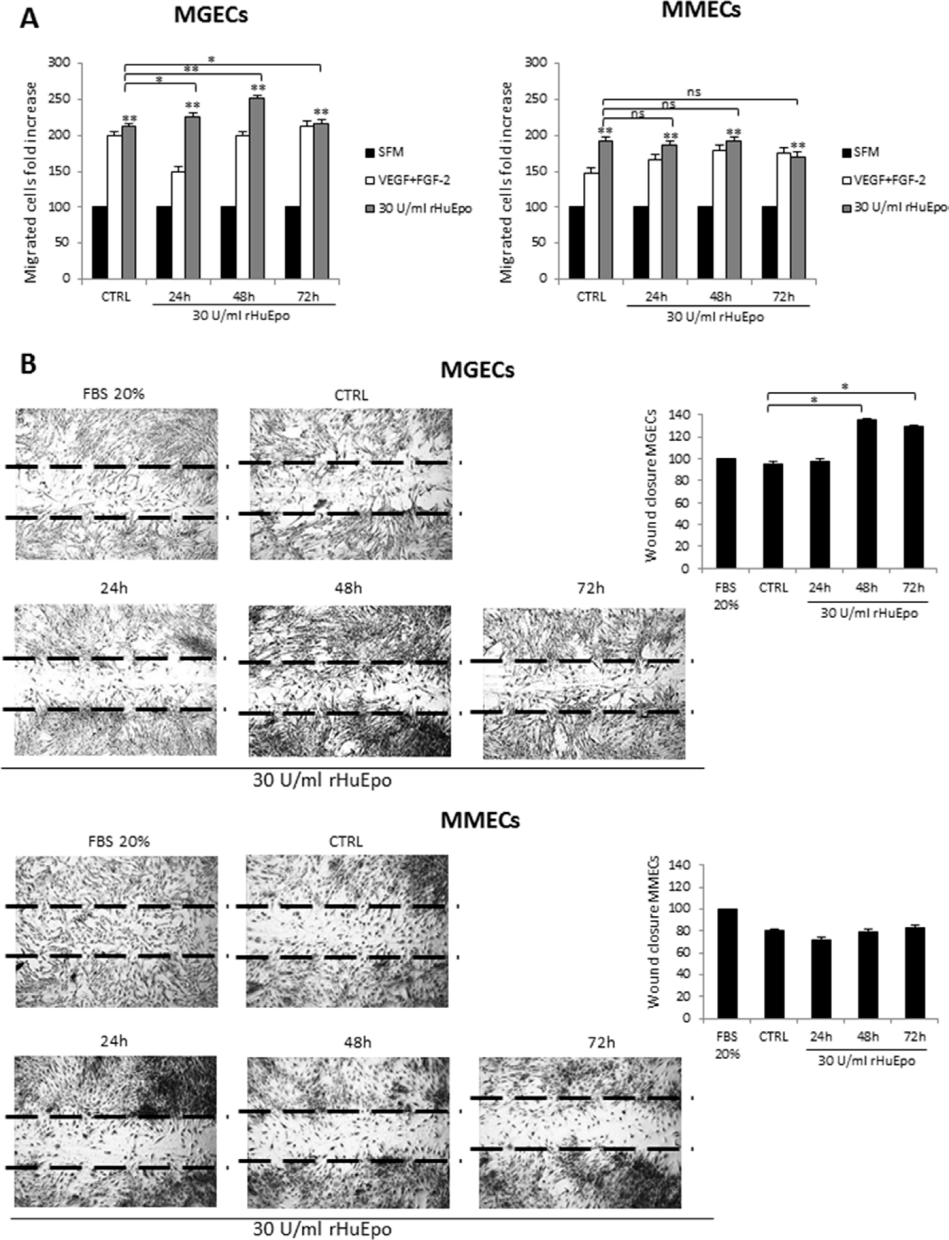
RHuEpo treatment enhances bone marrow endothelial cells motility *in vitro* (**A**) Boyden micro-chamber assay of MGECs (left) and MMECs (right) rHuEPO-treated for 24 h, 48 h and 72 h, fold increase of migrated cells in MGUS versus MM as mean ± SD of 8 MGUS and 6 MM patients; negative control arbitrary set as 100. (**B**) Wound healing assay of MGECs and MMECs rHuEPO-treated for 24, 48 and 72 h; representative images of the wound closure after 16 h from the scratch of 8 MGUS and 6 MM. Significances *P < 0.03 and **P < 0.003 by Wilcoxon signed-rank test.

To test chemo-induced migration of MGECs and MMECs treated with 30 U/ml of rHuEpo for 24, 48 and 72 h, Boyden chamber assay was used (Figure 5). Pre-treatment with rHuEpo did not change MGEC and MMEC migratory ability, but the addition of rHuEpo, as chemoattractant factor, at 24 and 48 h significantly enhanced MGEC induced migration more than of VEGF and FGF-2 agents. Conversely, MMECs did not response to chemoattractant activity of rHuEpo, except for not pre-treated cells (Figure 5A).

We also studied spontaneous cell migration through a scratch wound healing assay. MGECs closed more rapidly the wound when they were treated for 48 with 30 U/ml of rHuEpo. On the contrary, MMECs were not responsive to rHuEpo treatment (Figure 5B).

### RHuEpo stimulates angiogenesis *in vitro* and *in vivo*

**Figure 6:**
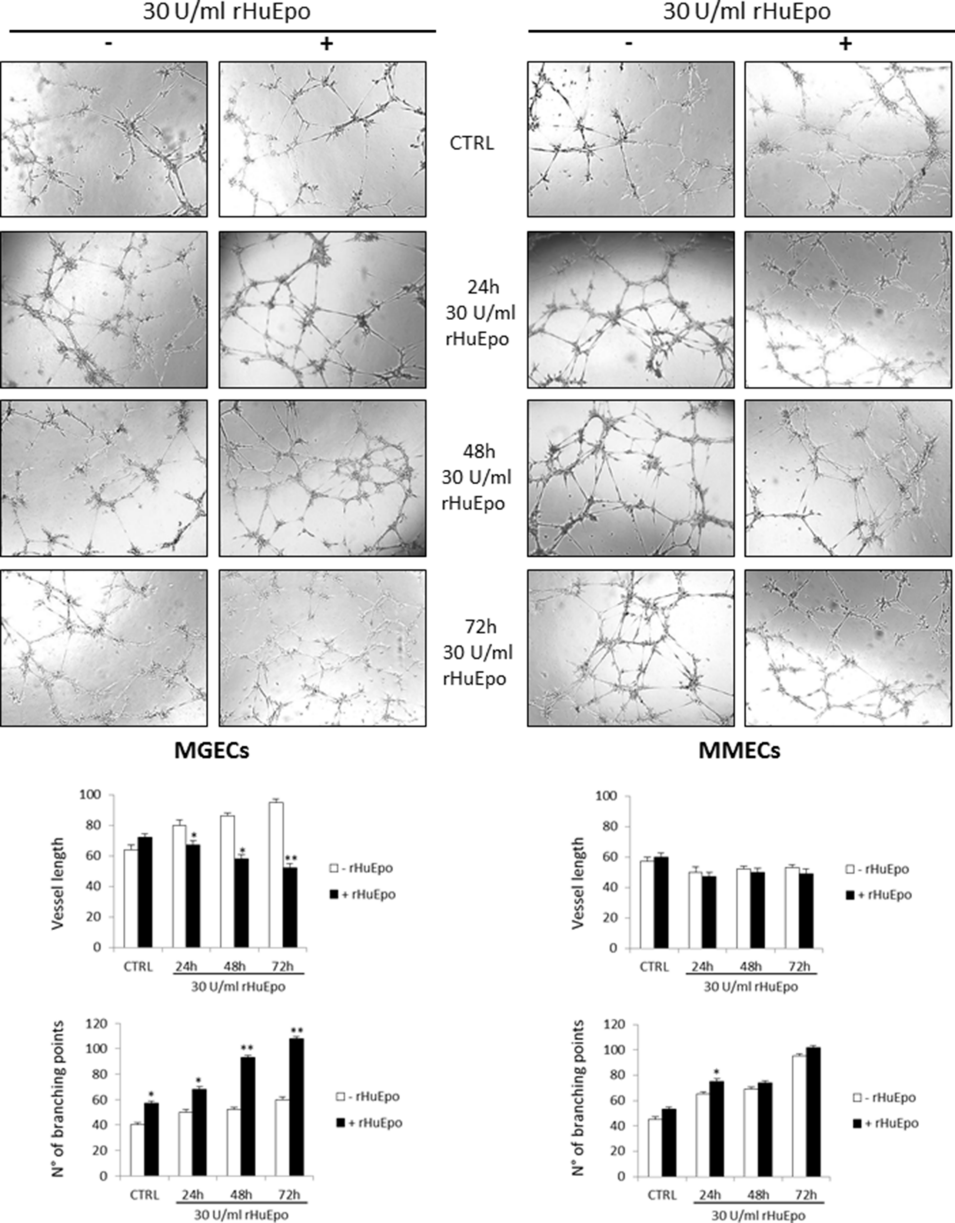
RHuEpo stimulates angiogenesis *in vitro* (**A**) Angiogenesis *in vitro* on Matrigel^©^. Images are representative of one MGUS and one MM patient out of total 8 MGUS and 6 MM analyzed by EVOS “Micron” image software. Significances *P < 0.03 and **P < 0.003 by Wilcoxon signed-rank test.

To verify if rHuEpo treatment enhances BMECs angiogenic ability, we performed *in vitro* and *in vivo* assays. As previously demonstrated (1, 15), MGECs are not able to form new capillary tubes *in vitro* on Matrigel^®^, but after pre-treatment with rHuEpo for 48 h, they acquired a strong angiogenic activity, forming branching, anastomosing tubes with multicentric junctions, and originating a meshwork of capillary-like structures (Figure 6). Otherwise, MMECs had intrinsic angiogenic ability *in vitro*. Pre-treatment with rHuEpo for 24–48 h did not lead to an increase in forming capillary-like structures, while pre-treatment for 72 h induced MMECs to form a higher number of vascular tubes assessed by number of branching points analysis (Figure 6).

**Figure 7:**
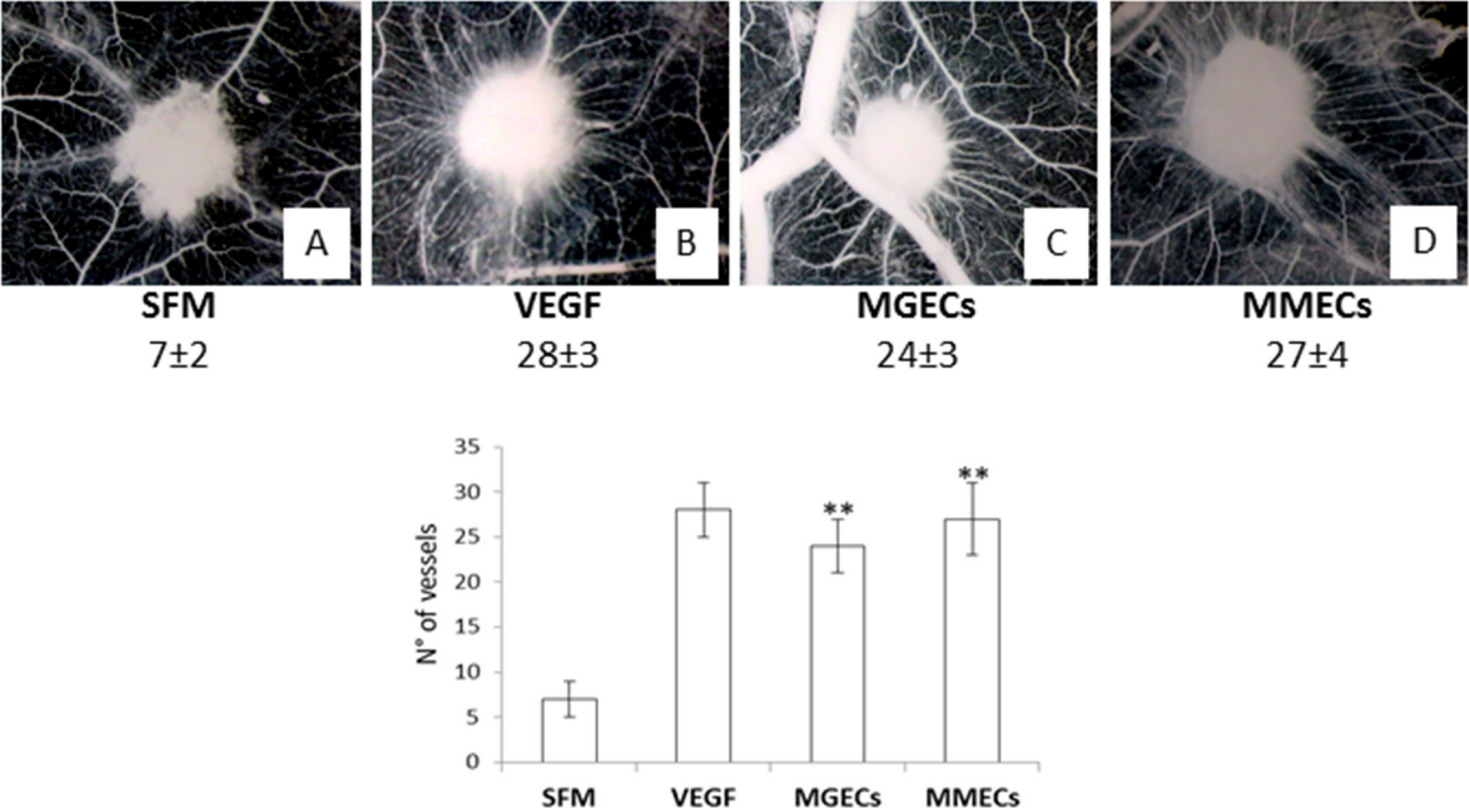
RHuEpo stimulates angiogenesis *in vivo* Macroscopic pictures of gelatin sponges soaked with serum free medium (SFM) alone (**A**) or supplemented with VEGF (**B**), or with 30 U/ml rHuEpo (C), or with the CM of MGECs and MMECs pre-treated with 30 U/ml rHuEpo (**C**, **D**), implanted on the chick embryo chorioallantoic membrane. Note numerous allantoic vessels developing radially towards the implants with the exception of the sponge adsorbed with SFM alone. Original magnification × 50. Significance *P* < 0.001 for MGECs and MMECs CM versus SFM alone.

We have previously demonstrated that MMECs and rHuEpo alone exerts a strong angiogenic activity in the CAM *in vivo* assay [1, 7]. Macroscopic observation of the CAMs showed that CM of MGECs and MMECs treated for 72 h with rHuEpo induced a strong angiogenic response (mean number of vessels: 24 ± 3 and 27 ± 4 respectively), characterized by newly formed capillaries spreading radially towards the sponges as compared to medium alone (mean number of vessels: 7 ± 2) (Figure 7).

## DISCUSSION

Anagnostou *et al.* demonstrated that Epo enhances the proliferation and migration of Human Umbilical Cord Vein ECs (HUVECs) as well as a strong positive EpoR staining of the *in vivo* vascular endothelium [10]. These evidences have been confirmed in Bovine Adrenal Capillary ECs (BACECs) [6, 11]. In 1999, we observed that rHuEpo induced an increased cell proliferation, the MMP-2 expression, and the differentiation into vascular tubes of the human EA.hy926 EC line *in vitro* [7]. Moreover, in the CAM assay, rHuEpo exerted an angiogenic activity comparable to that of FGF-2, and CAM's ECs expressed both EpoR and factor VIII-related antigen [7].

In this study, we show that EpoR is expressed by both MGECs and MMECs even though at a higher level in the first ones. Both EC types respond to rHuEpo in terms of cell proliferation, whereas other responses, including activation of JAK2/STAT5 and PI3K/Akt pathways, cell migration and capillarogenesis, are enhanced by Epo in MGECs, but not in MMECs. In addition, the CM of both Epo-treated cells induce a strong angiogenic response *in vivo* in the CAM assay, comparable to that of VEGF. In this context, Epo may be considered a key player in the “angiogenic switch” -occurring in the BM microenvironment during MM progression [12].

Epo is an endogenous stimulator of vessel growth during tumor progression through an autocrine/paracrine loops [2]. Tumor cells release increasing amounts of VEGF and placental growth factor (PlGF) in response to Epo [13]. In cerebral EC cultures, rHuEpo enhanced angiogenesis by increasing VEGF levels which were inhibited by anti-Epo antibody [14]. In the experimental model of femoral artery legation, blood flow recovery, activation of VEGF/VEGFR system, and mobilization of endothelial precursor cells (EPCs) were impaired in EpoR-null mice as compared to wild-type mice [15]. In the Lewis lung carcinoma xenograft model, subcutaneous administration of Epo promoted tumor growth through enhancement of angiogenesis [16]. In the dorsal skin-fold window chambers, the co-injection of Epo with mammary carcinoma cells stimulated tumor neovascularization and growth [17]. Finally, Epo/EpoR levels correlated with angiogenesis and progression in different human tumors [18–22].

Our results have also clinical implications. RHuEpo and Epo-stimulating agents (ESA), used to treat or to prevent anemia in oncological patients receiving chemotherapy including MM patients, negatively affected patient survival [23, 24]. Increased EpoR expression was identified as negative prognostic factor for overall survival and progression free survival [25, 26]. Moreover, Epo administration to patients with MM and myelodysplastic syndrome induced BM angiogenesis and further malignant transformation in plasma cell leukemia and acute monoblastic leukemia, respectively [27, 28]. ESA treatment of non-cancer related disease, including end-stage renal disease, could stimulate dormant tumor growth by mechanisms of promoting angiogenesis and tumor growth [29]. ESA may also facilitate tumor invasion and metastasis through angiogenesis, tumor cell mobilization, and upregulation of MMPs.

Administration of anti-Epo-antibody, soluble EpoR or an inhibitor of JAK2 resulted in a delay in tumor growth in a experimental model with rat mammary adenocarcinoma cells [30]. Similarly, injection of an anti-Epo antibody or the soluble form of EpoR into xenograft mice of uterine and ovarian cancer models reduced capillaries leading to tumor destruction [31]. Administration of putative anti-angiogenic agents targeting Epo/EpoR may be limited by development of anemia due to the inhibition of erythropoiesis. Otherwise, alleviation of anemia by systemic rHuEpo treatment can decrease hypoxia, enhances proliferation or survival of cancer cells, radiosensitivity of vessels and tumor perfusion by oxygen and chemotherapeutic agents, favouring their delivery [32].

Overall, our data suggest that Epo is involved in the regulation of angiogenic response occurring in MM through a direct effect on ECs, as well as on other cells of tumor microenvironment, including macrophages, as we have previously demonstrated [33]. MGECs are more responsive to Epo treatment than MMECs, probably because over-angiogenic phenotype of MMECs is already activated by their autocrine/paracrine loops occurring in the “angiogenic switch” from MGUS.

## MATERIALS AND METHODS

### Patients

Fifty patients fulfilling the International Myeloma Working Group diagnostic criteria for active MM (*n* = 27) and MGUS (*n* = 23) were studied. The study was approved by the local Ethics Committee of the University of Bari Medical School, and all patients provided their informed consent in accordance with the Declaration of Helsinki.

### Reagents

Recombinant Human Epo (rHuEpo, Eprex^®^, epoietin alfa) was provided by Janssen-Cilag (Sauderton, UK). Dulbecco's modified Eagle's medium (DMEM), heat-inactivated fetal bovine serum (FBS), antibiotic/antimycotic, tripsyn/EDTA and PBS without Ca^2+^ and Mg^2+^ were from Sigma-Aldrich (St Louis, MO).

### Isolation and characterization of MGECs and MMECs

Bone marrow aspirates were centrifuged on Ficoll-Hypaque (Pharmacia Biotech, Uppsala, Sweden) gradient centrifugation, and the separated mononuclear cells were left to adhere to 25-cm^2^ polystyrene flasks in complete medium (RPMI-1640 medium supplemented with 10% fetal calf serum [FCS] and 1% glutamine) for 2 h in culture conditions. Adherent cells were stromal cells, including ECs. To isolate ECs, stromal cells were harvested in trypsin/ethylendiaminotetraacetate (EDTA) solution [0.05/0.02% in phosphate-buffered saline (PBS)], washed twice with PBS, suspended in FCS-free medium (SFM), and immunodepleted of macrophages and possible residual plasma cells by a 30′ incubation in CD14 (a monocyte-macrophage marker) plus CD38 (a plasma cell and hematopoietic cell marker) monoclonal antibody (MoAb)–coated flasks (Immunotech, Coulter, Marseilles, France). Residual cells were suspended at 0.25 to 1 × 10^6^/mL in SFM and incubated for 30′ at 37°C with magnetic microbeads (Dynal, Oslo, Norway) at 0.15 to 0.5 × 10^6^/mL, respectively, coated with Ulex europaeus agglutinin-1 (UEA-1; Sigma Chemical, St Louis, MO), a lectin binding a specific receptor highly expressed by and restricted to ECs, in rotation. Microbeads with bound cells were recovered using a side-pool magnetic separation unit, transferred to 12-well plates in 3 mL complete medium/well, and left to migrate to the plate surface and grow. Fifteen to 20 days were needed to obtain 1 to 2 × 10^6^ cells per patient. The MGEC and MMEC population contained more than 95% factor VIII–related antigen (FVIII-RA)^+^ and CD31^+^ cells, as assessed by fluorescence activated cell sorting (FACS; FACS Calibur; Becton Dickinson, San Jose, CA). Contamination by macrophages and plasma cells was either ruled out or very insignificant, as evaluated by FACS with the CD14 and CD38 MoAbs, respectively, and by RT-PCR and Western blot for CD38. The trypan blue viability was more than 90%. To obtain their conditioned medium (CM), MGECs and MMECs at 90% confluence were cultured in SFM (approximately 1 × 10^6^/mL) for 24 h. CM were collected, sequentially centrifuged at 1200 and 12 000 rpm for 10′, respectively, filtered through sterilized 0.22 μm pore-size filters (Costar, Cambridge, MA), and stored at −80°C. FACS analysis for FVIII-RA, CD14, and CD38 was followed by RT-PCR and Western blot for FVIII-RA and CD38. RT-PCR was performed on 2 μg total RNA extracted with Trizol reagent (Invitrogen, Life Technologies, Carlsbad, CA) and reverse transcribed by Moloney murine leukemia virus reverse transcriptase (MMLV-RT; Invitrogen). Then, 1 μg cDNA was amplified by 22 to 35 cycles using human FVIII-RA primers (5′-GTTCGTCCTGGAAGGATCGG-3′-and 5′-CACTGACACCTGAGTGAGAC-3), human CD38 primers (5′-ACCCCGCCTGGAGCCCTATG-3′ and 5′-GCTAAAACAACCACAGCGACTGG-3′), and glyceraldehyde-3-phosphate dehydrogenase (GAPDH) control primers (5-CCCTCCAAAATCAAGTGGGG-3′ and 5′CGCCACAGTTTCCCGGAGGG-3′) (Invitrogen). Cell preparations with more than 95% FVIII-RA^+^ cells and no (or very insignificant) CD14^+^ and CD38^+^ cells were admitted to the sequence of tests.

### Real-time reverse transcription-polymerase chain reaction (real-time RT-PCR)

Total RNA was isolated using the RNeasy Mini kit (Qiagen Venlo, Netherlands) and reverse transcribed into total cDNA with the iScript cDNA Synthesis Kit (Bio-Rad Hercules, California, U.S.). Real-time RT PCR reactions were carried out using the “StepOne Real-Time RT-PCR System” (Applied Biosystems) and Taqman^®^ Real Time PCR technology (Applied Biosystems) [34]. The relative gene expression (fold change) was measured with the comparative threshold cycle (Ct) method using GAPDH as endogenous control and the 2^−ΔΔCt^ formula [35].

### Western blotting

Total protein lysate (35 μg) from MGECs and MMECs were separated on 4–12% NuPAGE^®^ gels (Invitrogen), electro-transferred to a polyvinylidene difluoride membrane (PerkinElmer Life Science Inc., Boston, MA) and immunoblotted with anti-Epo-R (M20, Santa Cruz Biotechnology Inc., Santa Cruz, CA), anti-pJAK2^(Tyr1007/1008)^, anti-JAK2, anti-pSTAT5^(Tyr694)^, anti-STAT5, anti-Akt, anti-pAkt^(Ser473)^ (Cell Signaling Technology Inc., Danvers, MA) and β-actin antibodies (Sigma-Aldrich). Then, the membrane was incubated with mouse and rabbit horseradish peroxidase–conjugated IgG (Bio-Rad, Hercules, California, U.S.). Immunoreactive bands were visualized by enhanced chemiluminescence (LiteAblot extend substrate, Euroclone) and the Gel Logic 1,500 Imaging System (Eastman Kodak Co., Rochester, NY), quantified with the Kodak Molecular Imaging Software, and the expression bands were quantify as arbitrary optical density units (OD).

### Immunofluorescence

Five × 10^3^ MMECs and MGECs were cultured on fibronectin-coated chamber slides (LabTek, Nalge Nunc International, Naperville, IL, USA), fixed (paraformaldehyde) and incubated with an anti-Epo-R rabbit polyclonal Ab (Santa Cruz Biotechnology Inc.), then with the anti-rabbit IgG-FITC Ab (Sigma-Aldrich). Nuclei were counterstained with4′,6-diamidino-2-phenylindole (DAPI), and mounting medium was used (Vectashield^®^, Vector, Burlingame, CA, USA). Pictures were acquired by an Axioplan-2 microscope (Carl Zeiss, Jena, Germany), and analyzed by the Leica Application Suite Advanced Fluorescence software (Leica Microsystems, Wetzlar, Germany).

### Cytokine measurement

Conditioned Media (CM) were obtained by seeding 2 × 10^5^ MGECs and MMECs and treating or not the cells with 30U/ml rHuEpo for 24 h, 48 h and 72 h. CM were collected and centrifuged at 1500 rpm at 4°C for 5′ to eliminate cell debris. Cytokines were measured by using Q-Plex^™^ Array Human Angiogenesis Antigen (Quansys Biosciences, Logan, Utah) allowing simultaneous quantification of the following cytokines in simple samples: angiopoietin-2 (ANG-2), FGF-2, hepatocyte growth factor (HGF), interleukin-8 (IL-8), platelet derived growth factor-BB (PDGF-BB), tissue inhibitor of matrix metalloproteinase-1 and -2 (TIMP-1, TIMP-2), tumor necrosis factor alpha (TNF-α) and VEGF according to the manufacturer's instructions. Secreted levels of cytokines were quantified through Q-View Software (Quansys Biosciences, Logan, Utah).

### Proliferation assay

MGECs and MMECs were seeded at 250 cells per well in 96-well plates in completed medium. After 24 h, medium was removed and the cells were washed twice with PBS 1X. Then, the cells were cultured in DMEM supplemented with 5% FBS and were treated with 15 U/ml, 30 U/ml and 60 U/ml rHuEpo for 24 h, 48 h and 72 h. The cell proliferation was evaluated with CellTiter-Glo^®^ Luminescent Cell Viability Assay (Promega Corporation, Madison, WI, USA) according to the manufacturer's instructions. The results are expressed as relative luminescence and this latter is directly proportional to cell number. Experiments were performed in quadruplicate.

### Chemotaxis assay

To evaluate MGECs and MMECs chemotaxis ability induced by rHuEpo treatment, cells (5 × 10^4^) were pre-treated and not pre-treated with 30 U/ml rHuEpo for 24, 48 and 72 h. The cells were tested in a Boyden microchamber on a polycarbonate membrane (Neuro Probe, Inc., Warwickshire, UK) pre-coated with 10 μg/ml fibronectin (Sigma-Aldrich) using SFM (as negative control), DMEM with 1.5% FBS added VEGF and FGF-2 (both 10 ng/ml; Miltenyi Biotec, Bergisch Gladbach, Germany) as chemoattractants (as positive control) and DMEM with 30U/ml rHuEpo. After 16 h at 37°C, the migrated cells were fixed, stained (Hema-Fast Kit, Exaxol Italia, Genova, Italy) and counted in five randomly chosen fields/well under a digital inverted light microscope EVOS (EuroClone) at 40 X.

### Wound healing assay

Eightx10^4^ MGECs and MMECs/well were seeded on fibronectin-coated (10 μg/ml; Sigma-Aldrich) 24-well plates and treated with 30 U/ml rHuEpo for 24, 48 and 72 h. FBS 20% was used as positive control and SFM alone as negative one. Cells were scraped as a “wound” with a pipette tip, and left to move into the wound for 16 h, then fixed and counted in at least three randomly chosen 10X wound fields on the EVOS microscope.

### *In vitro* capillarogenesis assay on matrigel

MGECs and MMECs pre-treated and not pre-treated with 30 U/ml rHuEpo for 24, 48 and 72 h were plated (2 × 10^4^) on 48-well plates coated with Matrigel (BD Biosciences) in SFM as control and with 30 U/ml rHuEpo. After 16 h the skeletonization of the mesh was followed by measurement of mesh areas and vessel length in three randomly chosen fields with the EVOS inverted microscope (EuroClone) at 40 × magnification.

### *In vivo* CAM angiogenesis assay

Fertilized chicken eggs were incubated at 37°C at constant humidity. On day 8, sterilized gelatin sponges adsorbed with SFM alone or supplemented with VEGF (100 ng/embryo) or with CM of MGECs and MMECs pre-treated for 24 h with 30 U/ml rHuEpo, were implanted on the top of the CAM, as previously described [36]. CAMs were examined daily until day 12 and photographed *in ovo* with a stereomicroscope. Blood vessels entering the sponges within the focal plane of the CAMs were counted by two observers in a double blind fashion at 50 × magnification.
